# Patient characteristics predict patency of early-cannulation arteriovenous grafts

**DOI:** 10.1038/s41598-021-87750-6

**Published:** 2021-05-24

**Authors:** David B. Kingsmore, Karen S. Stevenson, S. Richarz, Andrej Isaak, Andrew Jackson, Ram Kasthuri, Peter C. Thomson

**Affiliations:** 1 Renal and Transplant Surgery, Queen Elizabeth University Hospital, Glasgow, UK; 2 Vascular and Endovascular Surgery, University Hospital, Aarau-Basel, Switzerland; 3 Department of Nephrology, Queen Elizabeth University Hospital, Glasgow, UK; 4 Interventional Radiology, Queen Elizabeth University Hospital, Glasgow, UK; 5 Department of Vascular Surgery, Queen Elizabeth University Hospital, Glasgow, UK

**Keywords:** Haemodialysis, Renal replacement therapy

## Abstract

There is a new emphasis on tailoring appropriate vascular access for hemodialysis to patients and their life-plans, but there is little known about the optimal use of newer devices such as early-cannulation arteriovenous grafts (ecAVG), with studies utilising them in a wide variety of situations. The aim of this study was to determine if the outcome of ecAVG can be predicted by patient characteristics known pre-operatively. This retrospective analysis of 278 consecutive ecAVG with minimum one-year follow-up correlated functional patency with demographic data, renal history, renal replacement and vascular access history. On univariate analysis, aetiology of renal disease, indication for an ecAVG, the number of previous tunnelled central venous catheters (TCVC) prior to insertion of an ecAVG, peripheral vascular disease, and BMI were significant associates with functional patency. On multivariate analysis the number of previous TCVC, the presence of peripheral vascular disease and indication were independently associated with outcome after allowing for age, sex and BMI. When selecting for vascular access, understanding the clinical circumstances such as indication and previous vascular access can identify patients with differing outcomes. Importantly, strategies that result in TCVC exposure have an independent and cumulative association with decreasing long-term patency for subsequent ecAVG. As such, TCVC exposure is best avoided or minimised particularly when ecAVG can be considered.

## Introduction

Technical innovations in medical care have historically had a looser governance structure than drugs. The introduction of novel innovations into clinical practice is rarely based on large scale definitive trials. Instead the evidence gradually evolves from initial case-reports to case-series and more formal assessments that describe the practical aspects of their use. Subsequently larger case-series or more formal randomised trials focus on the optimal selection of cohorts, the wider description of outcomes, and the comparison to established products that may be industry sponsored.

Whilst the early aspects of this journey offer an opportunity to demonstrate the utility of a new and innovative product, they are equally open to significant limitations including: low statistical power that may result in limited success or failure having disproportionate effects; indication bias where the product is only used where the outcomes are likely to be better; selection bias in what data is selected for analysis; and publication bias which favours novelty and is less accepting of the outcomes from routine use or poor outcomes^1,2^. Thus the accommodation of innovation requires a critical volume of evidence prior to recognition of the potential role of novel devices which cannot be produced from meta-analysis of limited case-reports.

Early-cannulation arteriovenous grafts (ecAVG) are one such technical innovation that was produced as an evolution of traditional arteriovenous grafts (tAVG), but which now challenges established treatment paradigms. The choice of vascular access—arteriovenous fistula (AVF) or arteriovenous graft (tAVG), has been subject to many debates and trials for many years, with guidelines recommended limiting tAVG to those with limited or exhausted native AVF options^3,4^. A key weakness of tAVG is the lag time taken between implantation and subsequent use, during which alternative means of vascular access is required. This most commonly involves the use of the least preferred option—a tunnelled central venous catheter (TCVC) that incurs comparatively high morbidity and costs^5^. In contrast, ecAVG allow immediate use thus avoiding the requirement for a TCVC. Thus, ecAVG are not merely a refinement of established tAVG, but offer a new treatment paradigm for hemodialysis patients.

The published outcomes of ecAVG are highly variable and are typical of early reports of innovative products—small single-centre reports^6–15^, one industry supported multi-centre report^16^ and two randomised trials^10,12^. Despite the limited numbers of patients, there have been four reviews^17–20^. The published patencies vary widely, making it difficult to interpret their comparability to tAVG and their applicability.

These differences in outcome may partly relate to wide variation in both the population selected and the indication for which an ecAVG was used. However, no such association has ever been shown, perhaps as few centres have sufficient cases with detailed long-term follow-up with uniformity in the processes of care to demonstrate this. The recent KDOQI guidelines recommend a conceptual change with a wider recognition of the variation in the patient pathway, and an aim to match the choice of vascular access to anticipated patient need^21^. Data that could help select patients with better or poorer patencies would therefore be important when evaluating the use of ecAVG in this new paradigm.

Therefore, the aim of this study was to determine the factors that may predict long-term outcomes after ecAVG, especially with regard to those that are available to patients and clinicians at the time of deciding the vascular access strategy involving ecAVG.

## Methods

The outcomes of a consecutive cohort of all ecAVG (Gore Acuseal) including primary failures, placed in a single-centre (2/2012–12/2019) were retrospectively analysed from a prospectively recorded electronic patient record (ePR). ecAVG performed subsequent to this time were not included, as the short follow-up time precluded meaningful analysis of the primary outcome, loss of functional patency. During this time period there were 2661 AVF created (1090 at the wrist, 1507 in the elbow, and 64 miscellaneous including snuff-box and lower limb AVF). Data included demographics, complications and outcomes. Functional patency was used as the most meaningful outcome measure for patients and was defined as the interval between first cannulation and abandonment or the time of measurement of patency including occurrence of a censored event (death, elective change of modality, loss of follow-up)^22^. Other than 2 patients that were lost to follow-up after more than one year, all data was complete for the remaining patients. A multidisciplinary team meeting ran weekly with attendance from representatives of the vascular access specialist nursing team, interventional radiology, ultrasonography, nephrology and surgery, at which all imaging and planning was discussed. The technical aspects of AVG placement were consistent throughout the period with all data pertaining to one surgical team (DBK, KS, AJ). Where possible, all patients were treated with an anti-platelet (n = 194), with an anti-thrombotic (n = 66), or both (n = 18). The maintenance program was offered to all patients and over the first year of follow-up included: thrombectomy (1.9/patient/1000 dialysis days), angioplasty (2.1 / patient/1000 dialysis days), and stent-graft (1.4 /patient/1000 dialysis days). ecAVG were cannulated when required: 12% were cannulated on the day of surgery, and a further 21% on day one, with a median time to cannulation of 3 days. The primary infection rate was 3%, with secondary infection (AVG in use) occurring 0.27/1000 hdd, and bacteraemia rate of 0.08/1000 hdd *(in press).*

### Factors related to functional patency

Patient Factors: Details of patient co-morbidity were obtained from the documentation of past medical history and included co-existent disease (diabetes mellitus, previous coronary artery disease, stroke, hypertension, cardiac failure, peripheral vascular disease, malignancy), underlying aetiology of renal failure (diabetic nephropathy, glomerular nephritis, interstitial nephritis, unknown, multisystem diseases, other miscellaneous causes), smoking (active or not), body mass index (BMI, as a continuous and categorical variable), and medication (anti-coagulation, antiplatelets). The implantation site and configuration was recorded.

Renal Replacement Therapy (RRT): Data on previous RRT were recorded: time on RRT, previous RRT modality, previous vascular access (at time of operation for ecAVG, prior TCVC, prior AVF).

Indication for an ecAVG: The indications previously reported in case-series included poor native options for elective creation of an AVF, bilateral central vein stenosis or superior vena cava stenosis requiring lower limb ecAVG or HeRO (hemodialysis reliable outflow graft, Merit Medical), patient choice, TCVC complications including infection and dysfunction, salvage of failed AVF for thrombosis or aneurysm, and urgent—where dialysis was required before AVF creation had been achieved (Table 1). The primary indication for ecAVG was taken as the fundamental reason to receive an ecAVG rather than alternative vascular access. For example, an acutely occluded aneurysmal AVF that was excised and a replaced with an AVG in the same arm was categorised as AVF failure. However, if the AVG had been placed in the contra-lateral arm due that had no option for an AVF, then the indication was poor native options. Central vein stenosis as an indication was based on the need for either HeRO or lower limb ecAVG. Poor native options was based on the subjective surgical assessment based on several factors tha included ultrasound parameters (brachial artery (< 3 mm), severe distal calcification or incomplete palmer arch; cephalic vein < 2 mm at the wrist), or lack of adequate length (6 inches) of cannulatable conduit. An ecAVG was categorised as urgent if there was an acute need for dialysis with no vascular access ready. Thus a late-presenter would have the option of ecAVG or TCVC and may elect for an ecAVG and would be classified as ‘urgent’. If there was time before HD was required and an AVF was a viable option but the patient chose an ecAVG, then the indication would be patient choice. Importantly, the allocation to indication was made prior to the analysis of outcomes and obtained from the pre-operative documentation.

**Table 1:** Case-mix of case-reports of early-cannulation arteriovenous grafts.

Article	Demographic factors					Vascular access history				
	MeanAge	DM	BMI	Sex%male	RenalDisease	% Incident	Years HD	TCVC at op	AV at op	% Leg /HeRO
Aitken^7^ N = 37	42	97%	31	54	✕	11%	3.2	46%	8%	70%
Glickman^16^ N = 138	63	60%	30	49	✕	17%	2	89%	9%	0%
Maytham^11^ N = 55	64	38%		51	✕	20%			49%	0%
Tozzi^8^ N = 30	60	40%		60	✕		8.4mo	57%		10%
Aitken^12^ N = 60	54	37%	17%obese	53	✓	27%	3.4	22%	30%	3%
Chemla^23^ N = 16	56	47%		47	✕	0				0%
Chiang^24^ N = 45	52	60%		51	✕	62				0%
Schild^9^ N = 33	< 70	60%		48%	✕	0				0%
Lioupis^14^ N = 48	59	40%		65	✓	35				0%
Berard^15^ N = 46	63	39%	24	61%	✓	17%	1.3	74%	24%	22%
Scarrit^13^ N = 78	59	40%		65%	✕	35%				0%
Sutaria^6^ N = 141	61	46%		41	✕	26		51%	37%	2%8%

Article	Indication for AVG					Prev TCVC	Prev AVF	Factors in model		
	Urgent	AVF/G dys	TCVC dys	PNO	CVS					
Aitken^7^ N = 37	11%	8%	32%	9%	68%	65%	Mean 2	5/6		
Glickman^16^ N = 138	9%	7%	27%	20%	38%	53%	85%	5/6		
Maytham^11^ N = 55	0	25%/31%	0%	51%	0		25%	3/6		
Tozzi^8^ N = 30					0			2/6		
Aitken^12^ N = 60	12%	38%	21%		0	Mean 2	Mean 2	6/6		
Chemla^23^ N = 16						100%	100%	3/6		
Chiang^24^ N = 45		13%AVF 24%AVG					13%	2/6		
Schild^9^ N = 33							100%	3/6		
Lioupis^14^ N = 48							*100%*	3/6		
Berard^15^ N = 46		24%		74%		74%	43%	6/6		
Scarrit^13^ N = 78		19%					Median 1	3/6		
Sutaria^6^ N = 141	20%	33%	4%		8%			3/6		

*DM* diabetes mellitus, *BMI* body mass index, *Years HD* years on haemodialysis prior to ecAVG, *TCVC at op* presence of a TCVC at time of ecAVG operation, *AV at op* arteriovenous fistula at time of ecAVG operation, *leg/HeRO* lower limb or haemodialysis reliable outflow device used due to central venous stenosis. Shading, predictors in multi-variate model, Primary diagnosis, underlying cause of renal disease; *HD* hemodialysis, *TCVC* tunnelled central venous catheter, *Op* operation to implant ecAVG, *AV* arteriovenous fistula or graft, *LL* lower limb, *HeRO* hemodialysis reliable outflow device, *AVF/G Dys* dysfunction of an existing AVF or AVG, *TCVC Dys* dysfunction of a previously functioning TCVC, *PNO* poor native options, *CVS* central vein stenosis, Factors in model, number of factors documented in methods in the predictive model.

### Analysis

Initially the data was analysed to detect normality of distribution. Data that were not normally distributed included age and sex with greatly differing event rates. Age was included as a continuous variable in the multivariate analysis. As variables to be analysed were both quantitative and categorical, and with likely interaction, a Cox proportional Hazards analysis was performed. Predictors of poor outcome were sought using univariate analysis. There was no evidence of informative censoring as very few patients were lost to follow-up. The Wilcoxon test was used to test for significance rather than the log-rank due to the number of censored events.

To create the multivariate model, up to 10 predictors found significant on univariate analysis could be reliably fitted to the data, accepting the limitations to this particularly for time dependent outcomes^25,26^. A Cox proportional hazards model was performed based on continuous variable (age, number of TCVC) and categorical variables (sex, age in cohorts, smoking, presence/absence of co-morbid conditions, aetiology of ESRF, modality of vascular access at time of procedure, number of catheters, aetiology of ESRF, and indication).

The analysis was complicated due to the high level of censored events as it is assumed in the model that censored patients will have similar outcomes to non-censored which may or may not be true. The validity of the proportional-hazards assumption was made by making an interaction of the variable with time, with the significance of the time-dependent variable (T-cov) calculated when included in a Cox Model^27^. Schoenfeld residual analysis could not be performed to check the assumptions of proportionality due to the high intercurrent censored event rates which would be excluded using this method. There was a significant non-proportionality for sex (*p* < 0.04), with all other variables showing proportionality. The survival curves by sex demonstrated that up to 300 days, females had slightly poorer functional patency, whereas after this time period, males had poorer patency. Thus in the multi-variate analysis, a time-dependent model was used with this included.

All *p*-values were derived from two-tailed tests with *p* < 0.05 considered statistically significant. Data were analysed using SPSS software (IBM SPSS Statistics, Version 27).

The study was registered with the appropriate regional committee—The Renal Services Clinical Effectiveness Group. In the UK (NHS-Health Research Authority), formal research ethics approval was not required due to the retrospective, observational study of established practice within accepted guidelines with no additional tests performed for the purposes of research for which patient permission is included in the consent forms, with no patient identification. All methods were carried out in accordance with relevant guidelines and regulations.

## Results

### Outcomes

During the time-period studied, there were 2661 AVF created and 278 ecAVG implanted with 106 events (loss of ecAVG) and 137 censored events (death, elective change to other modality including transplantation). The mean follow-up was 481 days (s.d. 431 days), with a total exposure of 132,756 days.

### Cohort demographics

There were significant differences in the cohort demographics by age and sex (Table 2). Females comprised one-half of patients aged under 50 compared to only one quarter aged over 70, with the underlying primary renal disease more likely to be diabetic nephropathy compared to males (34% vs. 22%). Age had a significant impact on censored event rates—of the under 50′s, 25% were transplanted in follow-up, and 11% died, compared to the over 70′s in whom there were no transplants and 30% died. The type of vascular access at the time of operation also differed significantly by age: for patients < 50, they were more likely to have had no vascular access but were more likely to have had failing of an established modality of renal replacement history (RRT), compared to patients aged > 70 in whom one third had a previous AVF. There were no significant differences by age or sex for RRT (RRT at time of procedure, time on HD), vascular access history (the number of TCVC before the AVG, indication for an AVG) or for comorbidity (including BMI, cardiovascular disease or diabetes as a co-morbidity). Overall, 36% had a BMI of over 30.

**Table 2:** The distribution of case-mix and outcomes by age group and sex.

	Age group					
	< 50	51–70	71 +	Total		
**Sex**						
Female	61 (53%)	51 (43%)	10 (23%)	122 (44%)		
Male	54 (47%)	69 (57%)	33 (77%)	156 (56%)		
				*X*^2^ = 11. 44, p = 0.00		
**Outcome**						
Death	13 (11%)	13 (11%)	13 (30%)	39 (14%)		
Transplant	29 (25%)	24 (20%)	0	53 (19%)		
				*X*^2^ = 27.08, p = 0.04		
**Modality of RRT at time of AVG**						
None	31 (27%)	23 (17%)	3 (11%)	57 (20%)		
CVC	49 (42%)	70 (52%)	13 (48%)	132 (48%)		
AVF	12(10%)	26 (19%)	9 (33%)	47 (17%)		
PD/Tx/AVG	24 (21%)	16 (12%)	2 (7%)	42 (15%)		
				*X*^2^ = 17.463, p = 0.008		
Total	116	135	27	278		

	Sex				Total	
	Female		Male			
**Primary renal disease**						
Diabetes	42	34%	35	22%	77	28%
Glomerular nephritis	27	22%	48	31%	75	27%
IN	5	4%	7	4%	12	4%
Multisystem	3	2%	8	5%	11	4%
Other	40	33%	38	24%	78	28%
Unknown	5	4%	20	13%	25	9%
						*X*^2^ = 14.228, p = 0.014
Total	122	44%	156	56%	278	
						*X*^2^ = 27.08, p = 0.04

### Demographic impact on functional patency

**Figure 1:**
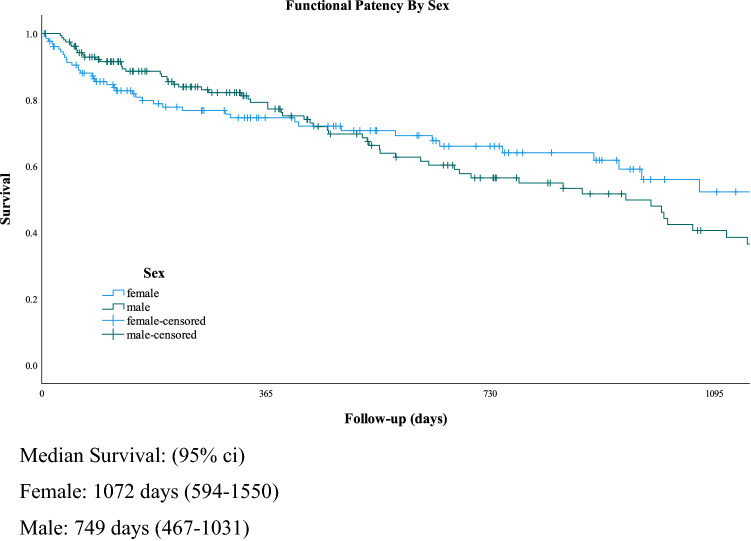
Kaplan–Meier survival curve of functional patency by sex.

On univariate analysis, the following factors were not significantly associated with functional patency: co-morbidity (coronary artery disease, cardiac failure, stroke, peripheral vascular disease, hypertension, malignancy, BMI and smoking), medication (anti-coagulants or anti-platelets); procedural factors (urgency of procedure, graft layout—upper vs. lower limb, straight vs. looped configuration). Although there was a consistent trend for age, sex and diabetes as adverse factors, and for hypertension to be a beneficial factor, none achieved significance on univariate analysis (Table 3). On analysis for a time-effect using the T-cov test, sex was found to be significant, with females having poorer functional patency in the first year, and better functional patency after one-year than males (Table 3, Fig. 1).

**Table 3:** Univariate analysis of risk of ecAVG survival (loss of functional patency): non-significant factors.

Factor	n	RHR	95% c.i	T-Cov	*X*^2^	*p* value
Stoke/TIA						
No	252					
Yes	22	0.739	0.385–1.421	0.077	0.829	0.364
Ischaemic heart disease						
No	196					
Yes	78	0.976	0.641–1.478	0.279	0.013	0.908
Smoking						
No	207					
Yes	67	0.909	0.583–1.417	0.280	0.177	0.674
Site						
Arm	193					
Leg	83	0.947	0.614–1.460	0.798	0.061	0.806
Hypertension						
No	120	1.00				
Yes	154	0.841	0.572–1.234	0.253	0.841	0.371
Age—continuous variable						
Mean 54.3	B = -0.997	(0.984–1.010)	0.377	0.199	0.655	
Sex						
Female	122	1.00				
Male	156	1.186	0.803–1.752	0.04	0.739	0.391
Diabetes						
No	175	1.00				
Yes	99	1.287	0.873–1.899	0.759	1.633	0.203
Medication						
Antiplatelet	212	1.00				
Warfarin	84	0.877	0.583–1.350	0.599	0.313	0.576
HD at time op						
PreDialysis	50	1.00				
HD/Tx/pd	224	0.936	0.567–1.543	0.428	0.068	0.795
Configuration						
Looped	154	1.00				
(Arm only)						
Straight	50	1.483	0.921–2.368	0.842	2.662	0.105
Time on RRT—continuous						
Median 1971		1.00 (1.00–1.00)	0.801	1.066	0.302	
Time on HD—continuous						
Median 1023		1.00 (1.00–1.00)	0.517	0.649	0.421

Functional patency was found to be significantly or of near significance related to five factors (Table 4): the number of prior TCVC (categorical and as continuous variables), the indication for an ecAVG; BMI; primary renal disease; and presence of peripheral artery disease. In particular, the use of a TCVC prior to getting an ecAVG was a significant predictor of poorer functional patency both as a categorical factor and a continuous factor with a 27% increased risk of loss of functional patency for each additional TCVC. Primary renal disease was associated with functional patency, with diabetes having significantly poorer and interstitial nephritis having significantly better functional patency. The indication for an ecAVG varied with ‘AVF replacement’ having significantly better and ‘poor native options’ having significantly poorer outcomes. BMI was directly correlated with functional patency, and the absence of peripheral arterial disease was associated with better patency.

**Table 4:** Univariate analysis of risk of ecAVG survival (loss of functional patency): significant/ near significance factors.

Factor	n	RR	95% c.i	T-cov	*X*^2^	*p* value
**No. of TCVC prior to ecAVG**						
0	113	1.00	Baseline			
1–2	100	1.159	0.738–1.820			0.521
3 +	62	1.656	1.031–2.660			0.037
				0.370	4.535	0.104
**No. TCVC—cnts**						
Mean 1.99		1.052	1.008–1.098	0.670	5.510	0.020
Indication for ecAVG*						
AVF Dys	48	1.00	Baseline			
CVS	59	1.648	0.903–3.007			0.103
Urgent	49	1.186	0.593–2.373			0.629
TCVC Dys	15	1.136	0.449–2.874			0.788
Failing RRT	15	0.797	0.296–2.146			0.654
Choice	7	0.677	0.157–2.913			0.600
Poor Options	50	2.302	1.259–4.207			0.007
LL (UL NNO)	33	1.234	0.571–2.668			0.593
				0.091	12.47	0.086
**BMI—cont**						
	Mean = 29.1, B = 0.021/unit	1.021	(0.996–1.048)	0.355	2.643	0.021
**Aetiology of renal failure**						
GN	73	1.00				
Diabetes	78	1.811	1.047–3.113			0.034
IN	11	0.500	0.067–3.723			0.499
Multi-system	12	2.027	0.763–5.382			0.156
Other	78	1.819	1.057–3.131			0.031
Unknown	24	1.505	0.721–3.139			0.276
				0.937	8.477	0.173
**Peripheral arterial disease**						
No	247					
Yes	26	1.696	0.944–3.048	0.875	3.193	0.074

*AVF/G Dys* dysfunction of an existing AVF or AVG, *TCVC Dys* dysfunction of a previously functioning TCVC, *PNO* poor native options, *CVS* central vein stenosis with HeRO or lower limb AVG, *LL* (UL NNO) lower limb AVG as upper limb had no native options, *GN* glomerular nephritis, *Diabetes* diabetes mellitus, *IN* interstitial nephritis.

### Multivariate model

**Figure 2:**
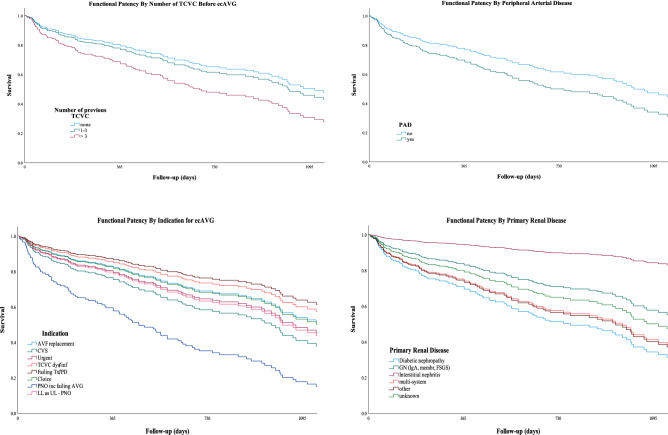
Functional patency survival curves by significant co-variables: body mass index, peripheral arterial disease, hypertension, primary renal diagnosis, and number of previous TCVC on univariate analysis.

A multivariate model was performed using the factors of near-significance and found to be highly significant (*X*^2^ = 24.078, *p* = 0.034, including age and BMI as continuous factors and sex as a time-dependent co-variable, Fig. 2, Table 5). Three factors were found to have independent significance (the number of prior TCVC, the indication for an ecAVG, and the presence of peripheral arterial disease) with the other factors not achieving significance (BMI, primary renal diagnosis). Interestingly, when number of TCVC was analysed as a continuous variable in the same model, there was a highly significant association (RR = 1.066, 1.011–1.124, *p* = 0.018) implying that for each additional TCVC, there was a corresponding higher risk of loss of functional patency of 6.6%.

**Table 5:** Multi-variable analysis of risk of loss of functional patency.

Factor	n	RHR	95% c.i	p value
**No. of TCVC (prior to ecAVG)**				
0	113	1.00	Baseline	
1–2	100	1.308	0.779–2.195	0.310
3 +	62	2.014	1.019–3.983	0.044
Indication for ecAVG*				
AVF Dys	48	1.00	Baseline	
CVS	59	1.573	0.779–3.176	0.207
Urgent	49	1.758	0.795–3.887	0.164
TCVC Dys	15	0.866	0.304–2.468	0.788
Failing RRT	15	0.975	0.307–3.099	0.965
Choice	7	0.908	0.198–4.159	0.901
Poor Options	50	2.823	1.429–5.557	0.003
LL (UL NNO)	33	1.536	0.660–3.573	0.319
**Peripheral arterial disease**				
Absent	230	1.00	Baseline	
Present	26	1.885	1.007–3.526	0.047

Model Significance: *X*^2^ = 24.078, p = 0.034.

Analysis performed after allowing for sex as a time-dependent variable, BMI as a continuous variable and age.

*AVF/G Dys* dysfunction of an existing AVF or AVG, *TCVC Dys* dysfunction of a previously functioning TCVC, *PNO* poor native options, *CVS* central vein stenosis with HeRO or lower limb AVG, *LL* (UL NNO) lower limb AVG as upper limb had no native options, *GN* glomerular nephritis, *Diabetes* diabetes mellitus, *IN* interstitial nephritis.

## Discussion

The recent KDOQi guidelines have emphasised the matching of choice of vascular access to individual end-stage kidney disease life-plan^21^. Central to this is an understanding of how the outcome of vascular access is related to patient factors and previous vascular access. This is the first paper to demonstrate that amongst the complex range of patient factors that may influence clinical decisions in use of ecAVG, there are three that are independently associated with outcome: the number of prior TCVC, the indication for which an ecAVG is employed, and the presence of peripheral vascular disease. Given that these data are available to clinicians and patients when making the selection of vascular access, it is possible to match the potential of various vascular access modalities against the patient need.

This analysis was stimulated by the highly variable case-mix and outcomes in case-series making meta-analysis inconclusive (Table 1). Few case-series presented complete data, only one had complete case-mix and indication, and thus these missing data could explain variations in reported patency. In the future, a more complete dataset may allow for a predictive model to be created. Even with the limitations of the current analysis, it is clear that some patients would be expected to have excellent patency (AVF replacement as indication, no previous TCVC, not obese) whereas others will do worse (poor native options, central vein stenosis, multiple prior TCVC, peripheral vascular disease and obese). Similarly, when comparing ecAVG to alternative forms of vascular access such as AVF, it is imperative that these factors are considered. In addition, this balance must be considered in context with the differences in the patient outcomes (death and transplantation) that determines the timescale for which vascular access may be required.

The number of potential predictors analysed in the formation of the multivariate model was determined before the analysis was performed on the basis of the 1 in 10 EPP ‘rule’ (events per predictor) accepting the limitations to this particularly for time dependent outcomes^25^. The predictors chosen (age, sex, number of prior TCVC, indication for AVG, underlying cause of ESRF) were the result of informal opinion from four consultants with vascular access specialist interest and over 45 years of combined clinical experience. Whilst it is accepted that this ‘rule’ has limitations particularly in model validation, refinement testing and in time-to-event analysis, for the first attempt to create a model to determine the relative impacts of these factors, this was thought reasonable^26^.

Whilst some factors analysed for predictive value were objective and easily recorded (age, BMI), others were subjective and may have had complex interactions. For example, patients with a short RRT history had few TCVC and few attempts at AVF and were thus more likely to have poor native options as the indication for an AVG. Poor native option itself is a very subjective assessment that is based more on the lack of good alternatives rather than a simple anatomical measurement. The volume of AVF procedures and the number of prior attempts indicates that the allocation to poor native options was truly reflective of a lack of alternative options. On the other hand, patients with a longer RRT time were more likely to have more TCVC, more attempts at AVF and the indication for an AVG was more likely to be CVS or to salvage an AVF. Thus, in developing the model we aimed to select predictors that were independent of each other where possible or modifiable.

Although the association with TCVC and outcome is dramatic, there are limitations to the implications of this. It was not practically possible with this dataset to determine the role of non-tunnelled CVC or the length of time for which TCVC were used. In addition, only one patient who subsequently developed post-operative hand swelling did not have a pre-operative venogram to ensure that non-clinically evident CVS was present. A subsequent investigation to understand the role of TCVC, number, site, location and duration particularly with reference to central vein stenosis may be possible in the future, but is out with the remit of this current analysis.

It is noteworthy that the definitions employed for indication were often not based on a single primary indication, but a collection of more than one secondary indication. We have however sought to determine the primary indication on the basis of the surgeon doing the procedure and this was documented prospectively, before the outcomes were known. It is difficult to entirely remove subjectivity from some of the definitions, whereas other aspects such as the number of TCVCs were entirely objective. The definition of poor native option was based on the surgeons perception based on small vessels on ultrasound and deficient conduit. Whilst these diameters were not fixed, it was a surgical decision based on the perceived patient need and likelihood of success of the AVF. Inevitably this means that there could be subjectivity in this selection, though all three implanting surgeons had similar views and practice. Furthermore, the routine practice of AVF creation in small vessels is demonstrated in the total number of procedures performed with only 10% of all vascular accesses created being prosthetic.

Some factors as expected did not impact on outcome. We found no difference by medication, in keeping with many meta-analysis, nor for any configuration or layout. Rarely were ecAVG refused though we have learned to be cautious with patients that may have increased risk of infection (active IV drug use, biological immunosuppression) or are pro-thrombotic (significant hypotension < 100 mmHg, active systemic infection). Rather than refuse ecAVG, our policy was to determine what would be required to sustain an ecAVG with consideration of optimising inflow volume, low resistance outflow, haemodynamic optimisation on haemodialysis and medication to prevent thrombosis. Thus for example, a patient who had repeated thrombosis of an upper limb ecAVG may be able to run a lower limb ecAVG with reduced anti-hypertensives and anti-coagulation.

Although this analysis benefits from a unified approach to surgery with a detailed comprehensive follow-up, there is a risk of over-analysis of a limited number of cases and events. However, for clinical decision making, a meaningful clinical endpoint must have sufficient importance to alter management. The findings that urgency and site of AVG do not alter long-term functional patency is consistent with other reports and supports the veracity of the dataset.

The methodology of ascertaining a correlation between functional patency and indication does not necessarily imply causality. We recognise that this simplified terminology summarises the summative endpoint of many complex processes. Thus, whilst the findings of predictors of outcome are interesting and novel, the only way of determining their veracity would be through prospective observation of pre-determined groups. Thus the findings of this study are best interpreted as supportive evidence for key elements of future prospective studies or randomised trials^28^.

There are four key messages from this analysis: firstly, that every TCVC carries with it an additive cumulative deleterious impact of subsequent patency. It is imperative therefore that early in the patient pathway consideration be given to TCVC-alternatives rather than persisting and have a dual impact—directly from additive TCVC and indirectly in changing the indication from dysfunctional TCVC to CVS or poor native options. Secondly, the outcome of an AVG must be considered and balanced against the alternatives for that patient, rather than the use of unadjusted aggregated outcomes. Thirdly, the optimal use of AVG is not limited to patients who have failed traditional vascular access and have no good options for a native AVF but should be considered an alternative for salvage of a failing AVF. Fourthly, patient centred medicine approaches demand that future trials should incorporate a structure for ensuring that these factors are recorded prospectively with sufficient power to allow meaningful group comparisons to more accurately understand how to optimally tailor treatment to patients.

## Data Availability

The datasets analysed during the current study are not publicly available due to patient confidentiality, but non-identifiable data may be made available from the corresponding author and institution on reasonable request and anonymisation.
